# Invasive Candida Infections in Neonatal Intensive Care Units: Risk Factors and New Insights in Prevention

**DOI:** 10.3390/pathogens13080660

**Published:** 2024-08-06

**Authors:** Niki Dermitzaki, Maria Baltogianni, Efrosini Tsekoura, Vasileios Giapros

**Affiliations:** 1 Neonatal Intensive Care Unit, School of Medicine, University of Ioannina, 45500 Ioannina, Greece; n.dermitzaki@uoi.gr (N.D.); mbalt@doctors.org.uk (M.B.); 2 Paediatric Department, Asklepieion Voula’s General Hospital, 16673 Athens, Greece; efitsek@gmail.com

**Keywords:** invasive candida infections, neonatal candidiasis, antifungal prophylaxis, fluconazole prophylaxis

## Abstract

Invasive Candida infections represent a significant cause of morbidity and mortality in neonatal intensive care units (NICUs), with a particular impact on preterm and low-birth-weight neonates. In addition to prematurity, several predisposing factors for Candida colonization and dissemination during NICU hospitalization have been identified, including prolonged exposure to broad-spectrum antibiotics, central venous catheters, parenteral nutrition, corticosteroids, H2 antagonist administration, and poor adherence to infection control measures. According to the literature, the implementation of antifungal prophylaxis, mainly fluconazole, in high-risk populations has proven to be an effective strategy in reducing the incidence of fungal infections. This review aims to provide an overview of risk factors for invasive Candida infections and current perspectives regarding antifungal prophylaxis use. Recognizing and reducing people’s exposure to these modifiable risk factors, in conjunction with the administration of antifungal prophylaxis, has been demonstrated to be an effective method for preventing invasive candidiasis in susceptible neonatal populations.

## 1. Introduction

### 1.1. Epidemiology

Invasive *Candida* infections represent a significant cause of morbidity and mortality in the neonatal intensive care unit (NICU) population, with a reported incidence of 0.5–2% [1,2]. Preterm and very-low-birth-weight (VLBW) neonates are at a particularly high risk of developing invasive *Candida* infections, with the incidence being inversely correlated with gestational age (GA) and birth weight (BW) [3]. The rate of these infections varies significantly between NICUs and different geographic locations. The incidence is notably higher in developing countries than in developed countries [4,5,6,7,8,9,10,11]. As the most susceptible neonatal population, in extremely low-birth-weight neonates (ELBW neonates), an incidence of 2 to 20% has been described [3,12,13,14,15,16,17,18]. A recent prospective study involving 10,501 neonates of GA < 29 weeks with late-onset sepsis (LOS) reported that fungal microorganisms were detected in 5.1% of cases; however, in the subgroup of neonates of GA < 23 weeks, the proportion of fungal infections rose to 10% [2].

An increase in the incidence of invasive fungal infections was observed in the 1980s and 1990s, which was attributed to improved survival among preterm infants. However, a gradual reduction has been reported over the last two decades, probably due to the increased use of antifungal prophylaxis and empirical antifungal treatment, and a reduced use of antimicrobial agents [11,17,19,20]. A retrospective study of data from 322 NICUs over a 14-year period revealed a decline in the incidence of invasive neonatal fungal infections. The annual incidence decreased from 3.6 to 1.4 per 1000 patients among the study population and this reduction was more pronounced as the birth weight declined (from 82.7 to 23.8 per 1000 infants with BW < 750 g) [17].

Invasive fungal infections in neonates, especially VLBW neonates, are associated with significant morbidity and mortality. A mortality rate of 30% has been described in preterm and low-birth-weight neonatal populations [11,13,20,21,22]. Death or adverse neurodevelopmental outcomes were observed in 73% of a cohort of 320 ELBW neonates with IC [13]. One retrospective Canadian study compared the outcomes of extremely preterm neonates with IC, infection by other pathogens, and neonates with no history of infection and concluded that mortality and adverse neurological outcomes were significantly higher in the group of neonates with IC than in the other two groups [21].

### 1.2. Microbiology

*Candida* spp. species constitute a common component of the human mycobiome with pathogenic potential [23]. A range of virulence factors contribute to *Candida* spp.’s ability to cause systemic infection, including epithelial adhesion and invasion, biofilm formation, and the secretion of hydrolytic enzymes [24,25]. Invasive infections are often associated with the formation of biofilms on tissues or implanted medical devices [23,24,26]. The treatment of *Candida* biofilms represents a significant therapeutic challenge. The eradication of these infections is frequently difficult to achieve, which is further compounded by the fact that biofilms act as a reservoir for the dissemination and prolongation of infection [23,26]. A common feature of biofilms is that they frequently comprise polymicrobial communities in which bacteria and fungi coexist [27]. Concomitant infection by *Candida albicans* and *Staphylococcus aureus* is often observed in the neonatal population [26]. It has been demonstrated that *Candida albicans* can be found in biofilms with a variety of bacteria, comprising *Staphylococcus epidermidis*, *Acinetobacter baumannii*, *Pseudomonas aeruginosa*, and various *Streptococcus* species. It is noteworthy that even anaerobic organisms, such as *Bacteroides fragilis* and *Clostridium perfringens*, are capable of proliferation in oxygen-rich environments when present within a *Candida* biofilm [26]. These multi-species infections are associated with significant morbidity and mortality. In these infections, *Candida* and bacterial species act synergistically, which results in protection between the involved species and the exacerbation of drug resistance. Treatment is complex, and a multi-drug approach is necessary [26,28].

*Candida albicans* is the most frequently isolated strain in neonatal invasive infections, followed by *Candida parapsilosis* and, less commonly, *Candida glabrata*, *Candida tropicalis*, *Candida krusei*, and the recently emerged *Candida auris* [11,21,29,30,31,32]. The prevalence of different strains varies according to geographic location, with a higher rate of *non-albicans* species observed in low- and middle-income countries [32,33]. It is noteworthy that recent cohort studies from geographic regions other than Europe and Northern America, such as China, India, Brazil, and South Africa, have documented a shift towards a predominance of *non-albicans Candida* strains [4,7,8,9,34,35].

### 1.3. Colonization

Neonates admitted to the NICU, particularly VLBW neonates, represent a population with a relatively high frequency of *Candida* colonization [1,36]. Colonization by *Candida* species represents an initial necessary step in the pathogenesis of invasive infection, providing a repository for subsequent dissemination under predisposing conditions [37,38].

*Candida* species colonize the skin, the gastrointestinal tract, and the genitourinary system. Acquisition of colonization in the neonatal population can be either vertical or horizontal [20,38,39]. A number of studies have identified vaginal delivery as a risk factor for neonatal colonization [39,40,41,42,43,44,45,46,47,48]. The incidence of vaginal *Candida* colonization is known to increase during pregnancy, particularly in the last trimester, and the reported prevalence varies considerably in the literature, ranging from 5.6 to 69.2% [39,49]. *Candida albicans*, the most common *Candida* strain causing vaginal colonization during pregnancy, is the predominant strain isolated in cases of vertical transmission [39,50]. In a cohort of 102 preterm and VLBW neonates delivered either vaginally or by cesarean section, 12.8% of the vaginally delivered neonates were detected to be colonized within the first week of life, whereas no colonization was identified in the cesarean-delivered group. It is noteworthy that all mothers of the colonized preterm neonates were also colonized by the same species. The risk of neonatal colonization was found to be correlated with the duration of premature rupture of membranes (PROM) and inversely correlated with birth weight [39]. A higher risk of perinatal colonization as the gestational age and birth weight decline has been described in several studies [44,51]. A recent study used molecular techniques to compare Candida isolated from maternal oral mucosa during pregnancy with oral isolates from their colonized infants. The results demonstrated a robust genetic similarity between *Candida* spp. species. Furthermore, the study revealed a significantly higher incidence of Candida colonization in infants born to mothers with an increased density of oral Candida colonization and a strong association between vertical Candida acquisition and maternal plaque index scores [50].

In addition to transmission during the birth process, horizontal acquisition of *Candida* spp. can occur from the NICU environment. The most common sources of transmission are the hands of healthcare providers, contaminated equipment, or intravenous preparations [20,44,52]. This mode of transmission represents the primary source of *Candida parapsilosis* infection, as it has been reported as the most common strain isolated from the hands of healthcare providers. Conversely, *C*. *parapsilosis* is rarely implicated in vertical transmission [20,39,52].

Colonization is considered to be the initial step in the pathogenesis of IC. However, colonization does not necessarily lead to dissemination and systematic infection [38]. It is estimated that more than 60% of VLBW neonates are colonized during the first month of their NICU hospitalization [36]. The incidence of invasive fungal infections in colonized VLBW neonates ranges from 8 to 23% [39,44,53,54]. Therefore, the recognition of additional predisposing factors for disseminated disease is crucial as it could aid in the identification of high-risk neonates.

## 2. Risk Factors and Prevention

The aim of this narrative review is to summarize the risk factors for invasive Candida infections and current perspectives regarding antifungal prophylaxis use. The PubMed and Google Scholar databases were searched for relevant studies on risk factors of invasive candidiasis and antifungal prophylaxis. The following keywords were used: “neonatal invasive candidiasis”, “neonate”, “invasive candidiasis risk factors”, “fluconazole prophylaxis”, “nystatin prophylaxis”, and “antifungal prophylaxis”. Peer-reviewed studies published up to July 2024 were included, particularly randomized control trials, systematic reviews, narrative reviews, and observational studies. Additionally, the reference lists of the retrieved articles were examined to identify any relevant studies that might have been missed in the initial search.

### 2.1. Risk Factors

#### 2.1.1. Prematurity and Low Birth Weight

Invasive *Candida* infections are rarely observed in neonates with a normal birth weight. In a large retrospective study including neonates from 302 NICUs with a birth weight above 1500 g, IC was reported in 0.06% of neonates [55]. Notably, the risk of systemic *Candida* infection increases with decreasing gestational age and birth weight [56]. Predisposing factors, in addition to their prolonged stay in the NICU and increased need for invasive procedures, include their relative immunodeficiency and immature skin and mucosal barriers [1,57,58]. ELBW neonates are at increased risk of developing disseminated *Candida* infection and rates of up to 20% have been reported [18]. Even in this high-risk, extremely preterm population, the prevalence varies according to gestational age. In a multicenter retrospective cohort study involving 26 NICUs in Canada, Zhou et al. reported an increasing incidence of IC with decreasing gestational age. They observed IC in 0.2% of neonates born at 28 weeks’ gestation and 4.6% of neonates born at 22–23 weeks’ gestation (21).

#### 2.1.2. Type and Number of Colonization Sites

Several studies have indicated a correlation between the type and number of colonization sites and the risk of progression to IC [39,44,53,54]. In a cohort of 201 VLBW neonates colonized by *Candida* spp. at any time during their hospitalization, colonization of the central venous catheter (CVC) and colonization in more than three sites were identified as independent risk factors for the progression to IC [53]. Mahieu et al. reported that no cases of IC were detected in neonates colonized at the skin exclusively. However, the prevalence of IC in neonates with gastrointestinal colonization was 16.6%, and when both aforementioned sites were colonized, the incidence was 41.7% [44]. Manzoni et al. reported a threefold increase in the incidence of IC in neonates colonized in more than three sites. Moreover, they observed that certain sites of colonization, such as urine and catheters, are associated with a fourfold-increased risk of IC compared to other sites, including the skin, nasopharynx secretions, and gastric aspirates [54].

#### 2.1.3. Broad-Spectrum Antibiotics

Broad-spectrum antibiotics represent a significant modifiable risk factor for IC [20]. It is well established that antibiotics suppress the normal gastrointestinal bacterial flora, thereby limiting its competitive action that would normally prevent the overgrowth of *Candida*. This effect is probably more pronounced in the immature gut microbiota of neonates [59]. The increased density of *Candida* species predisposes them to translocation across the intestinal epithelium and dissemination [20,38,56,59]. Several studies have identified an association between the use of broad-spectrum antibiotics, more commonly third-generation cephalosporins and carbapenems, and the development of systemic *Candida* infections in neonates [13,18,36,55,56,60,61]. Among different NICUs, the incidence of IC in ELBW neonates has been shown to correlate with the average use of broad-spectrum antibiotics per neonate, predominantly third-generation cephalosporins [18]. Moreover, it has been reported that in neonates with a birth weight < 1500 g, a 2.9–7.3% decrease in episodes of IC is observed for every 10% reduction in broad-spectrum antibiotic use [17]. A recent multicenter case–control study identified antibiotic exposure as a risk factor for IC in VLBW neonates and demonstrated that an antibiotic use rate increased by 10% and an increase in exposure with each additional day, in particular exposure to third-generation cephalosporins and carbapenems, were associated with an increased incidence of IC [62]. Furthermore, Eisi et al. identified prolonged antibiotic exposure as an independent risk factor for deep-tissue invasion of *Candida* in a population of neonates with IC [63]. It is therefore important to implement an antimicrobial stewardship strategy in NICUs, limiting the use of antibiotics and the duration of antibiotic therapy and encouraging the use of narrower-spectrum antibiotics, particularly when there is no evidence of central nervous system involvement [64,65].

#### 2.1.4. Central Venous Catheters

Central venous catheters penetrate epithelial barriers, allowing for the invasion of *Candida* in normally sterile sites. In addition, the capacity of *Candida* spp. species to adhere to and form biofilms on devices protects them from the host’s immune response and antifungal agents [20,56,61]. Therefore, central venous catheters, widely used in VLBW neonates during their NICU stay, represent a significant risk factor for developing systemic candidiasis [13,20,56,60,61,66,67,68]. It has been reported that the risk of IC increases with each additional day that a central catheter remains in place [68]. A recent retrospective study in a cohort of very preterm and VLBW neonates demonstrated that the risk of colonization of peripherally inserted central catheter (PICC) lines was associated with the duration of antibiotics and parenteral nutrition, and the administration of corticosteroids postnatally [69]. Furthermore, due to the ability of drug-resistant biofilm formation, the delayed removal of catheters in ELBW neonates with IC has been identified as a factor that prolongs the duration of fungemia and increases the risk of end-organ involvement, adverse neurodevelopmental outcomes, and mortality [13,61].

#### 2.1.5. Corticosteroids

Corticosteroids are widely used in NICUs to reduce pulmonary morbidity among preterm neonates by preventing or managing chronic lung disease [70]. A recent survey of 397 NICUs in Europe revealed that the majority of NICUs administer corticosteroids during the second or third week of life to facilitate extubation and/or to prevent bronchopulmonary dysplasia (BPD) in high-risk neonates regardless of the mode of respiratory support [71]. It is well established that corticosteroids exert immunosuppressive effects, including a decreased circulating T-lymphocyte count, suppressed cytokine responses, and impaired cell-mediated immunity [20,72]. Several studies have indicated a potential association between the administration of steroids and the risk of IC in preterm neonates [20,72,73,74,75]. A retrospective case–control study of neonates with a birth weight < 1250 g identified dexamethasone administration during the first two weeks of life as a risk factor for systemic candidiasis [72]. However, no association was observed between postnatal corticosteroid use and an increased risk of deep-tissue candidiasis in a neonatal cohort with IC [63].

#### 2.1.6. Histamine Type 2 Receptor (H2) Antagonists

H2 antagonists act by inhibiting gastric acid secretion. Alkalization modifies the commensal bacterial flora and promotes gastric colonization and the proliferation of Gram-negative bacteria and fungi, and subsequently their translocation across the gastrointestinal tract. Moreover, H2 antagonists can exert immunomodulatory effects by influencing neutrophil activity [20,38,61,66,76]. Saiman et al., in a prospective multi-center study, reported that the administration of H2 antagonists in neonates was associated with a twofold-increased risk of developing systemic candidiasis [66].

#### 2.1.7. Gastrointestinal Pathologies

Gastrointestinal pathologies, including necrotizing enterocolitis (NEC), congenital malformations, and prior abdominal surgeries, represent significant risk factors for the development of systemic fungal infections. The disruption of the mucosal and epithelial intestinal barriers allows the translocation of colonizing *Candida* in the gastrointestinal tract and into the bloodstream [11,12,20,68]. The association between gastrointestinal pathologies and IC in the neonatal population has been demonstrated in several studies [12,41,55,60,68,77].

#### 2.1.8. Parenteral Nutrition

Parenteral nutrition represents a fundamental aspect of the care provided to ELBW neonates during their stay in the NICU [78]. However, it is a recognized predisposing factor for bacterial and fungal infections in the neonatal population [13,56,66,73,79,80,81]. De Susa et al. observed an association between the prolonged administration of parenteral nutrition and late-onset sepsis in VLBW neonates [82].

Specifically, lipid emulsion has been shown to facilitate the proliferation of *Candida* and its capacity to form biofilms on indwelling catheters [83,84]. Furthermore, contamination during the preparation of parenteral nutrition solution has been reported as a potential causative factor in *Candida* outbreaks in NICUs [85]. Saiman et al. identified parenteral nutrition as a predisposing factor for systematic candidemia, and this association was observed independently of central venous catheter use [66]. Moreover, a recent retrospective study by Menezes et al. recognized parenteral nutrition as an independent risk factor for neonatal IC [86].

### 2.2. Prevention

In addition to their relative immunodeficiency and immature skin and mucosal barriers, preterm and low-birth-weight neonates represent a population frequently exposed to a variety of predisposing factors that pose to them a particular risk of acquiring fungal infections during their prolonged stay in NICU. Given the potential adverse effects of *Candida* infections on survival and neurodevelopment in this immature population, it is evident that primary prevention represents a crucial aspect.

#### 2.2.1. Fluconazole Prophylaxis

In the prevention of candidiasis, in addition to minimizing the exposure of the most susceptible neonates to potential predisposing factors, the prophylactic administration of antifungal agents represents a widely used approach [87]. The most commonly used agent is fluconazole, a long half-life azole with good tissue penetration [88,89]. Fluconazole prophylaxis has been studied since the 1990s in immunocompromised adult and pediatric populations [90]. The first randomized control trials (RCTs) regarding the efficacy of fluconazole prophylaxis in susceptible neonatal populations were published in 2001 and reported potentiated results regarding its utility in the prevention of colonization and invasive infections [91,92]. Since then, several RCTs and retrospective studies with historical control cohorts have examined its efficacy, the optimal dosing regimen, its adverse effects, and the associated resistance patterns [37] (Table 1). A recent prospective weekly point prevalence study conducted in 27 European NICUs revealed that fluconazole was the most widely used antifungal agent for prophylaxis in high-risk neonates, being employed in over 98% of cases [93].

##### Efficacy

Neonates have a unique opportunity to reap the beneficial effects of fluconazole prophylaxis, which differs from those observed in pediatric and adult populations. At birth, the majority of VLBW neonates are not yet colonized or have low-density colonization. This offers an opportunity for antifungal medication to act either by preventing colonization or reducing the proliferation of the yeast in already-colonized patients [90,104]. It is noteworthy that in the absence of antifungal prophylaxis, up to 60% of VLBW neonates will be colonized by Candida until the third week of life [104]. Several studies have indicated a significant reduction in the incidence of colonization in vulnerable neonatal populations following the administration of fluconazole prophylaxis [91,92,94,97,101]. In a recent systematic review and meta-analysis by Anaraki et al., which included seven studies with the outcome of colonization rate, a significant decrease in the *Candida* colonization rate was reported in VLBW neonates who received fluconazole prophylaxis [105].

The efficacy of fluconazole prophylaxis regarding reducing the incidence of systematic *Candidiasis* in high-risk neonates has been recognized in a number of RCTs and retrospective studies [36,91,94,95,97,98,100,102,106,107]. It is worth noting that in the majority of these studies, the incidence of IC in the control group is high (up to 45%), which is higher than that typically observed in developed countries at present. Consequently, the benefit of fluconazole prophylaxis may be overestimated and less pronounced in NICUs with a lower incidence of systemic candidiasis [108,109]. It has been proposed that in settings with an IC incidence of approximately 16%, the number needed to treat to achieve benefit (NNTB) is 11 [110]. However, in a multi-center RCT of neonates with a birth weight < 750 g with a low rate of probable or definitive IC in the control group (9%), although no significant difference was observed regarding the composite outcome of death or IC between the two groups, the rate of IC was significantly lower in the fluconazole-administered group. This suggests that high-risk neonates in low-risk settings might benefit from antifungal prophylaxis [99].

In other studies, despite the lower colonization rate among neonates receiving fluconazole prophylaxis, the incidence of IC was not observed to differ in the prophylaxis group. Two of these studies reported a low incidence of IC in the control group (4%), while the other, conducted in India, found that non-albicans *Candida* spp. were responsible for almost all invasive infections [92,96,101].

A recent meta-analysis by Xie et al. demonstrated a significant reduction in the risk of IC and in-hospital mortality associated with fluconazole prophylaxis (RR = 0.37, *p* = 0.0006 and RR = 0.75, *p* = 0.004, respectively) [111].

Current guidelines released by the Infectious Diseases Societies of North America (IDSA) and the European Society of Clinical Microbiology and Infectious Diseases (ESCMID) recommend routine fluconazole prophylaxis in ELBW neonates in NICUs with a high incidence of IC (>10%) [112,113]. In settings where the incidence of IC is lower (>2%), an individualized approach with risk factor estimation is advised [113].

##### Dosing

The optimal dosing regimen for fluconazole prophylaxis remains inconclusive. The dosing interval varies substantially among studies, ranging from daily to biweekly administration [108]. In an RCT, Kaufman et al. reported that twice-weekly administration is as effective for *Candida* prophylaxis in ELBW neonates as more frequent dosing [80]. Furthermore, a pharmacokinetic analysis demonstrated that adequate serum levels can be achieved with biweekly administration [114]. Less frequent dosing is associated with lower fluconazole exposure and probably lessens the risk of drug resistance [108,115].

With regard to the dosage, the majority of studies employ a dose of either 3 mg/kg or 6 mg/kg [116]. Manzoni et al., in a multi-center RCT, demonstrated that both of these doses are equivalent in their effectiveness in preventing IC [97]. Moreover, in their meta-analysis, Leonart et al. reported that the effectiveness of the prophylaxis was not affected by the dose administered (3, 4, or 6 mg/kg/dose). The latter authors concluded that, given the greater potential for adverse effects, antifungal resistance, and higher cost associated with higher doses, a lower dose (3 mg/kg/dose) should be recommended [116].

Another aspect of fluconazole prophylaxis administration that varies among studies is the cessation time. In most studies, prophylaxis in ELBW neonates is continued until the chronological age of 28 or 42 days (Table 1). The results of one meta-analysis indicate that prophylaxis until the 42nd postnatal day may be more efficacious [117]. The IDSA and ESCMID guidelines both recommend a dosage of 3 to 6 mg/kg administered intravenously or orally twice weekly for 6 weeks [112,113].

##### Adverse Effects

Several studies have evaluated the potential risk of adverse effects associated with fluconazole, including abnormal liver tests, cholestasis, sepsis, and NEC. These studies have found no significant association between fluconazole and these adverse effects [36,91,92,94,97,98,99,100]. Nevertheless, although fluconazole appears to be safe for neonates, it is advisable to use lower doses to avoid potential adverse events and drug reactions. In a prospective study, Kaufman et al. evaluated neurodevelopmental outcomes among VLBW neonates who received fluconazole prophylaxis at the age of 8 to 10 years old and observed no association between fluconazole and adverse neurodevelopment [118].

##### Resistance

The potential emergence or predominance of *Candida* spp. strains with native or acquired resistance to fluconazole represents a significant concern regarding the strategy of fluconazole prophylaxis in high-risk neonates [106,119]. Studies in susceptible adult populations have demonstrated the emergence of resistant strains following treatment with antifungal prophylaxis [119]. Although still inconclusive, the data from studies in neonatal populations are more reassuring, probably because the development of resistance is associated with the length of drug exposure, the cumulative dose, and the proportion of admitted patients in the unit receiving prophylaxis concomitantly. Previous RCTs have demonstrated a lack of emergence of resistant strains [91,92,120]. A meta-analysis conducted by Ericson et al. indicated that there was no significant difference in the resistant strains isolated from prophylaxis-treated and control groups [108]. Moreover, in a single-center retrospective study from Italy, no change in fungal ecology was detected over 20 years, including 4 years before the implementation of the fluconazole prophylaxis strategy and 16 years after [119].

A recent multi-center RCT in ELBW neonates reported a clinically insignificant higher minimum inhibitory concentration (MIC) of isolated *Candida* colonization species after fluconazole prophylaxis administration [106]. Furthermore, in a recent retrospective study by Zhang et al., although no completely resistant species were detected, isolates with significantly higher MICs were observed in the group of neonates that received prophylaxis. This is likely due to the high proportion of *Candida glabrata*, a strain with a higher propensity for resistance development [36]. Lee et al. observed a higher, though not statistically significant, incidence of IC fluconazole-resistant *C*. *parapsilosis* in ELBW infants following five years of routine fluconazole prophylaxis [101].

The aforementioned evidence suggests that fluconazole prophylaxis appears to be a safe and effective strategy for reducing IC in high-risk neonates. However, an alternative approach to antifungal prophylaxis has been suggested in cases of colonization with azole-resistant *Candida* species, such as *Candida auris*, or NICUs with a high prevalence of resistant strains. In these settings, micafungin prophylaxis may be a suitable option [30,37,121]. Micafungin is the sole echinocandin approved by both the Food and Drug Administration (FDA) and the European Medicine Agency (EMA) for use in infants. However, its use is limited due to the potential hepatotoxicity and the paucity of data regarding pharmacokinetics in the neonatal population [122].

#### 2.2.2. Nonabsorbable Antifungal Agents

Nonabsorbable antifungal agents, such as nystatin and miconazole oral gel, are not systemically absorbed and aim to reduce the density of fungal colonization in the gastrointestinal tract and its subsequent dissemination [113,123].

A number of RCTs and prospective studies have reported a reduced incidence of IC in VLBW and ELBW populations after oral nystatin prophylaxis administration in comparison to those administered no prophylaxis [123,124,125,126] (Table 2). One meta-analysis indicated that nystatin prophylaxis was associated with a significant reduction in IC. However, the presence of methodological weaknesses and heterogeneity among the included studies precludes a conclusive interpretation of these results [127]. Furthermore, a recent meta-analysis of five RCTs, comprising a total of 1750 neonates, has demonstrated that nystatin prophylaxis is associated with a significant reduction in the rates of fungal colonization and IC. Nevertheless, no benefit was observed in terms of mortality and the length of hospitalization when compared to placebo or no-drug treatments. However, the authors have acknowledged certain methodological limitations among their included studies [128]. Oral nystatin and oral or intravenous fluconazole treatments have demonstrated similar efficacy in RCTs that include VLBW and ELBW neonates [129,130]. The current IDSA and ESCMID guidelines recommend the use of oral nystatin 100,000 UI q8 as an alternative to fluconazole for the prophylaxis of fungal infections in susceptible neonates when fluconazole is unavailable or if resistant species have been isolated [112,113].

A significant limitation to the use of oral nystatin is that VLBW and ELBW neonates frequently present with medical conditions that preclude the use of oral preparations, including hemodynamic instability, feeding intolerance, and gastrointestinal diseases [37,112]. Moreover, a potential increased risk of NEC is associated with nystatin administration due to its hyperosmolar composition [127].

The efficacy of miconazole oral gel as an antifungal prophylaxis has been previously investigated in an RCT involving 600 neonates. The incidence of *Candida* colonization was significantly lower in the miconazole prophylaxis group compared to the placebo group. However, no significant difference was observed in the incidence of IC, which can be attributed, at least in part, to the low rates of IC in the study cohort (2% and 2.6% in the miconazole and placebo groups, respectively) [131].

#### 2.2.3. Probiotics

The potential benefits of probiotic supplementation in VLBW neonates have been the subject of numerous RCTs and observational studies over the last two decades [132,133]. Several recent systematic reviews and meta-analyses have reported a decreased incidence of NEC, late-onset sepsis, and mortality with the use of probiotics [134,135,136,137].

It has been postulated that probiotics may inhibit the colonization and proliferation of *Candida* in the intestinal tract. This is thought to occur through a competition for colonization sites and through alteration of the mucosal barrier’s permeability, immune-mediated responses, and active metabolite production [138,139,140,141]. It has been proposed that probiotics inhibit biofilm production at early stages [141]. Alshaiki et al. observed a notable reduction in the abundance of *Candida* spp. in the intestinal mycobiome of ELBW infants who were supplemented with probiotics [142]. A meta-analysis of seven RCTs indicated that probiotics may have a beneficial effect in relation to reducing Candida colonization in preterm neonates. However, data regarding the efficacy of probiotics in preventing IC in NICUs were inconclusive [139]. Two RCTs in VLBW populations compared two different strains of probiotics, *Lactobacillus reuteri* and *Saccharomyces boulardii*, to nystatin prophylaxis and both demonstrated similar efficacy regarding colonization and IC reduction. Additionally, in both trials, the probiotic group demonstrated a reduced incidence of bacterial sepsis and feeding intolerance [143,144]. Conversely, a recent multi-center cohort study demonstrated that the incidence of IC was significantly higher in preterm neonates exposed to probiotics than in the control group [145].

Nevertheless, concerns regarding the safety of probiotic administration, particularly regarding certain strains, especially in ELBW neonates, preclude their universal administration in this population [132,133,146]. In conclusion, the effect of probiotics in the prevention of IC in preterm neonates and the optimal strain, dosage, and duration of administration remain controversial [37].

#### 2.2.4. Lactoferrin

Bovine lactoferrin, a glycoprotein known to enhance the maturation of the intestinal barrier and the immunological properties of the intestinal mucosa, has been demonstrated to exert antifungal activities by disrupting the fungal cell membrane [147,148]. In a multi-center RCT, Manzoni et al. observed a significantly reduced incidence of IC in VLBW neonates receiving bovine lactoferrin alone or in combination with Lactobacillus rhamnosus GG, in comparison to the placebo group. The rate of fungal colonization in both groups was found to be similar [147]. Nevertheless, the results of recent RCTs have not identified a reduction in the incidence of late-onset sepsis in neonates who received lactoferrin [149,150,151]. The largest RCT to date, the ELFIN trial, did not demonstrate any benefit regarding IC with lactoferrin administration [152]. However, a recent systematic review of nine RCTs reported a reduced incidence of fungal sepsis in preterm neonates who received lactoferrin or lactoferrin plus probiotics compared to a placebo. No adverse effects associated with lactoferrin were reported in the included studies [153].

According to the current Infectious Diseases Societies of North America (IDSA) and the European Society of Clinical Microbiology and Infectious Diseases (ESCMID) guidelines, bovine lactoferrin (100 mg/d), administered alone or in combination with Lactobacillus rhamnosus, may be efficacious as a prophylactic measure for IC [112,113]. However, recent studies have used doses based on body weight (150–200 mg/kg/day) [149,150,151].

## 3. Conclusions

Invasive *Candida* infections in NICUs represent a significant challenge. Preterm and low-birth-weight neonates are at particularly high risk of developing disseminated diseases. The potentially detrimental effects of IC in this vulnerable population concerning their survival and neurodevelopment underscore the need to recognize and avoid modifiable risk factors, such as the extensive use of broad-spectrum antibiotics, central venous catheter use, corticosteroid administration, and poor compliance with hygiene measures. The use of antifungal prophylaxis with fluconazole in high-risk neonatal populations, especially in settings with a high prevalence of fungal infections, has been proven to be an efficacious measure to reduce the incidence of Candida colonization and its progression to disseminated disease. In conclusion, the development of strategies to minimize exposure to risk factors and the integration of such strategies in clinical practice in NICUs, along with the use of antifungal prophylaxis in cases where it is indicated, has been demonstrated to be an effective approach for reducing the incidence of IC.

## Figures and Tables

**Table 1:** Studies investigating the efficacy of fluconazole prophylaxis.

Author	Study	Population	Dosing	Colonization	IC	Mortality Attributable to Candida	Overall Mortality
Kicklighter, 2001 [92]	RCT	100 VLBW (50 fluconazole, 50 placebo)	6 mg/kg/72 h 7 d, 6 mg/kg/24 h 8–28 d	15.1% vs. 60% (*p* = 0.0005)	4% vs. 4%	ND	ND
Kaufman, 2001 [91]	RCT	100 ELBW (50 fluconazole, 50 placebo)	3 mg/kg/72 h 1–2 wk, 3 mg/kg/48 h 3–4 wk, 3 mg/kg/24 h 5–6 wk (iv)	22% vs. 60% (*p* = 0.002)	0% vs. 20% (*p* = 0.008)	ND	8% vs. 20% (*p* = 0.22)
Manzoni, 2006 [94]	Pre–post cohort study	465 (225 fluconazole, 240 control)	6 mg/kg/72 h for 7 d, 6 mg/kg/48 h until 30 d VLBW, 45 d ELBW or discharge (iv/per os)	26.4% vs. 71.9% ELBW (*p* < 0.0001) 22% vs. 35% VLBW (*p* = 0.01)	4.4% vs. 16.7% (*p* < 0.0001)	0% vs. 1.7% (*p* = 0.7)	ND
Aghai, 2006 [95]	Pre–post cohort study	177 ELBW (140 fluconazole, 137 control)	3 mg/kg/72 h 1–2 wk, 3 mg/kg/48 h 3–4 wk, 3 mg/kg/24 h 5–6 wk (iv)	ND	0% vs. 6.6% (*p* = 0.006)	ND	25.7% vs. 39.4% (*p* = 0.02)
Parikhi, 2007 [96]	RCT	120 VLBW (60 fluconazole, 60 placebo)	3 mg/kg/72 h 7 d, 3 mg/kg/24 h 8–28 d	19% vs. 50% (*p* < 0.001)	26.7% vs. 25% (*p* = 0.835)	ND	ND
Manzoni, 2007 [97]	RCT	363 VLBW (112 fluconazole 6 mg/kg, 104 fluconazole 3 mg/kg, 106 placebo)	6 mg/kg/72 h 2 wk, 6 mg/kg/48 h until 4 wk VLBW, 6 wk ELBW, or 3 mg/kg/72 h 2 wk, 3 mg/kg/48 h until 4 wk VLBW, 6 wk ELBW	9.8% vs. 29.3% (*p* < 0.001) 7.7% vs. 29.3% (*p* < 0.001)	2.7% vs. 13.2% (*p* = 0.005) 3.3% vs. 13.2% (*p* = 0.02)	0% vs. 1.9% (*p* = 0.23) 0% vs. 1.9% (*p* = 0.57)	8% vs. 9.4% (*p* = 0.81) 8.7% vs. 9.4% (*p* = 1)
Aziz, 2010 [98]	Pre–post cohort study	262 ELBW (163 fluconazole, 99 control)	3 mg/kg/72 h 1–2 wk, 3 mg/kg/48 h 3–4 wk, 3 mg/kg/24 h 5–6 wk, or 3 mg/kg biweekly	ND	1.8% vs. 7.1% (*p* = 0.045)	ND	9.2% vs. 5.1% (*p* > 0.05)
Benjamin, 2014 [99]	RCT	362 BW < 750 g (188 fluconazole, 175 placebo)	6 mg/kg biweekly until 42 d (iv/per os)	ND	3% vs. 9% (*p* = 0.02)	ND	14% vs. 14% (*p* = 0.98)
Kirpal, 2015 [100]	RCT	75 VLBW (38 fluconazole group, 37 placebo)	6 mg/kg/48 h for 7 d, 6 mg/kg/24 h until 28 d or discharge (iv)	ND	21% vs. 43.2% (*p* < 0.05)	2.6% vs. 18.9% (*p* < 0.05)	ND
Lee, 2016 [101]	Pre–post cohort study	423 ELBW (264 fluconazole, 159 control)	3 mg/kg biweekly for 4 wk (iv or per os)	33.9% vs. 59.1% (*p* < 0.001)	5.0% vs. 4.4% (*p* = 0.80)	3.2% vs. 11.5% (*p* = 0.32)	11.7% vs. 16.4% (*p* = 0.18)
Silva-Rios, 2019 [102]	Pre–post cohort study	893 neonates (484 ELBW universal fluconazole prophylaxis, 409 VLBW targeted prophylaxis)	3 mg/kg/72 h (iv or per os)	ND	3.7% vs. 7.1% (*p* = 0.04)	0% vs. 17.1% (*p* = 0.015)	ND
Zhang, 2021 [36]	Pre–post cohort study	196 VLBW (113 fluconazole, 83 control)	6 mg/kg biweekly, 4 wk VLBW, 6 wk ELBW or discharge (iv)	ND	15.9% vs. 45.8% (*p* < 0.001)	2% vs. 4% (*p* = 0.69)	ND
Valenzuela-Stutman, 2023 [103]	Pre–post cohort study	353 VLBW [125 intervention cohort (53 ELBW universal fluconazole prophylaxis, 14 VLBW targeted prophylaxis, 58 VLBW no prophylaxis), 220 control cohort]	3 mg/kg biweekly	ND	2.4% vs. 7.8% (*p* = 0.05)	ND	*p* > 0.05

VLBW: very-low-birth-weight infants; ELBW: extremely low-birth-weight infants, iv: intravenous, ND: no data.

**Table 2:** Studies investigating the efficacy of nystatin prophylaxis.

Author	Type of Study	Population	Colonization	IC	Mortality
Sims, 1998 [124]	RCT	67 VLBW (33 nystatin, 34 control)	12% vs. 44% (*p* < 0.01)	6% vs. 32% (*p* < 0.001)	12% vs. 20% (*p* < 0.05)
Howell, 2009 [126]	Prospective multicenter surveillance	12,607 VLBW (7738 nystatin, 4868 control)	ND	0.54% vs. 1.23% (*p* < 0.01)	ND
Aydemir, 2010 [129]	RCT	278 VLBW (94 nystatin, 93 fluconazole, 91 placebo)	11.7% vs. 10.8% vs. 42.9% (*p* < 0.01)	4.3% vs. 3.2% vs. 16.5% (*p* < 0.01)	8.5% vs. 8.4% vs. 12.1% (*p* = 0.64)
Mersal, 2013 [130]	RCT	57 VPT < 30 wks and/or <1200 g (24 nystatin, 33 fluconazole)	12% vs. 8%	0% vs. 0%	
Rundjan, 2020 [123]	RCT	95 VLBW/VPT (47 nystatin, 48 placebo)	29.8% vs. 56.3% (*p* = 0.009)	0% vs. 10.4% (*p* = 0.056)	14.9% vs. 18.8% (*p* = 0.616)

VLBW: very-low-birth-weight infants; VPT: very preterm infants, ND: no data.
